# Treatment with Mesenchymal Stromal Cells Overexpressing Fas-Ligand Ameliorates Acute Graft-versus-Host Disease in Mice

**DOI:** 10.3390/ijms23010534

**Published:** 2022-01-04

**Authors:** Andrei Mircea Vacaru, Ana-Maria Mazilu, Madalina Dumitrescu, Ioana Madalina Fenyo, Anca Violeta Gafencu, Ana-Maria Vacaru

**Affiliations:** Gene Regulation and Molecular Therapies Laboratory, Institute of Cellular Biology and Pathology “Nicolae Simionescu”, 050568 Bucharest, Romania; anamazilu6@gmail.com (A.-M.M.); madalina.dumitrescu@icbp.ro (M.D.); madalina.fenyo@icbp.ro (I.M.F.); anca.gafencu@icbp.ro (A.V.G.); ana.vacaru@icbp.ro (A.-M.V.)

**Keywords:** graft versus host disease, mesenchymal stromal cells, FasL, bone marrow transplantation, immunosuppression

## Abstract

Allogeneic hematopoietic cell transplantation (allo-HCT) has the potential to cure malignant and non-malignant hematological disorders, but because of the serious side effects of this intervention its applications are limited to a restricted number of diseases. Graft-versus-host disease (GvHD) is the most frequent complication and the leading cause of mortality and morbidity following allo-HCT. It results from the attack of the transplanted T cells from the graft against the cells of the recipient. There is no clear treatment for this severe complication. Due to their immunomodulatory properties, mesenchymal stromal cells (MSC) have been proposed to treat GvHD, but the results did not meet expectations. We have previously showed that the immunomodulatory effect of the MSC was significantly enhanced through adenoviral-mediated overexpression of FasL. In this study, we have tested the properties of FasL-overexpressing MSC in vivo, in a mouse model for acute GvHD. We found that treatment with FasL-overexpressing MSC delayed the onset of the disease and increased survival of the mice.

## 1. Introduction

Acute graft-versus-host disease (aGvHD) is the most prevalent complication after allogeneic hematopoietic cell transplantation (allo-HCT). Depending on the type of transplantation, the incidence of aGvHD varies between 35% and 80% of transplants. First-line treatment is represented by corticosteroids, but up to 50% of the patients do not respond to therapy and have bad prognosis because of the absence of a universally established second-line treatment [1]. Establishing a treatment for this disease would expand the clinical applications of allo-HCT that has the potential to cure autoimmune diseases, such as type-1 diabetes, multiple sclerosis, or lupus, that are currently incurable. aGvHD is triggered by T cells from the graft that recognize the cells of the recipient as foreign. The most damage is inflicted to three main targets: the skin, the gastrointestinal tract, and the liver. The severity of aGvHD results from the degree of involvement of the three main target organs [2].

Mesenchymal stromal cells (MSC) are multipotent cells that can be isolated from adult tissues such as bone marrow, adipose tissue, dental pulp, inner organs, and peripheral blood, as well as from neonatal sources such as placenta and umbilical cord. In vitro, they can be induced to form a number of mesodermal cell types such as adipocytes, chondrocytes, and osteocytes. MSC have been long considered very good candidates for cell-based therapies because of their multipotent nature, however, part of the excitement has vanished when their limited in vivo differentiation properties became apparent [3,4]. The hype was reignited once it turned out that MSC have immunomodulatory potential. Through secreted factors and cytokines, they are able to impair the proliferation of T and B cells, dendritic cells, and natural killer cells. MSC are also capable to induce regulatory T cells (Treg) [5]. Interestingly, parts of their immunomodulatory capacities are observed for apoptotic or dead MSC as well [6]. Due to their immunomodulatory properties, MSC have been initially considered good cell-therapy candidates for the prevention and the treatment of GvHD in patients undergoing HCT. While the results regarding the prophylactic and therapeutic effects of MSC for GvHD showed that MSC alone are not potent enough to cure GvHD, it is clear that they have beneficial effects and represent a good adjuvant therapy [7].

The first apoptosis signal ligand (FasL) is a member of the TNF ligand family. Its specific interaction with the receptor, Fas, results in the activation of a caspase cascade that initiates apoptosis [8]. The Fas-FasL-mediated cell death plays a major role in immune homeostasis: it is required for the deletion of autoreactive lymphocytes during negative selection of T cells in the thymus [9] or during the physiological immune response in the periphery [10]. FasL-induced signaling is required not only for the death of T cells, but also for deletion of autoreactive B cells, B cell somatic hypermutation, cytotoxicity of NK and CD8^+^ T cells, apoptosis of endothelial cells, regulation of myeloid suppressor cells’ turnover, and activation of macrophages functions against infections [11]. FasL is exposed on the cell membrane for a few minutes from secretion until shedding [12]. Only the membrane-bound FasL is able to induce apoptosis while soluble FasL activates different signaling cascades [13]. There are studies suggesting that FasL promotes the survival of immature hematopoietic progenitor cells [14] and at low concentrations it promotes the proliferation of bone marrow-derived MSC [15].

MSC from bone marrow express low levels of FasL and mediate Fas-dependent cell apoptosis [16,17,18]. It was also demonstrated that the endogenous FasL expressed by MSC was able to transiently increase the apoptosis of T cells via the FasL/Fas pathway, leading to immune tolerance [19]. We have shown in a recent study that overexpression of FasL following adenovirus transduction enhances the immunomodulatory properties of the MSC. In the current study, we have set to investigate the potential of bone marrow-derived MSC overexpressing FasL (named killer MSC or kMSC) to treat aGvHD. We report here that kMSC obtained from bone marrow of BALB/c mice have superior immunomodulatory activity against stimulated splenocytes compared with naïve MSC and are able to delay the setup of the disease and to extend the survival in a mouse model of aGvHD.

## 2. Results

### 2.1. Generation of Killer MSC

**Figure 1:**
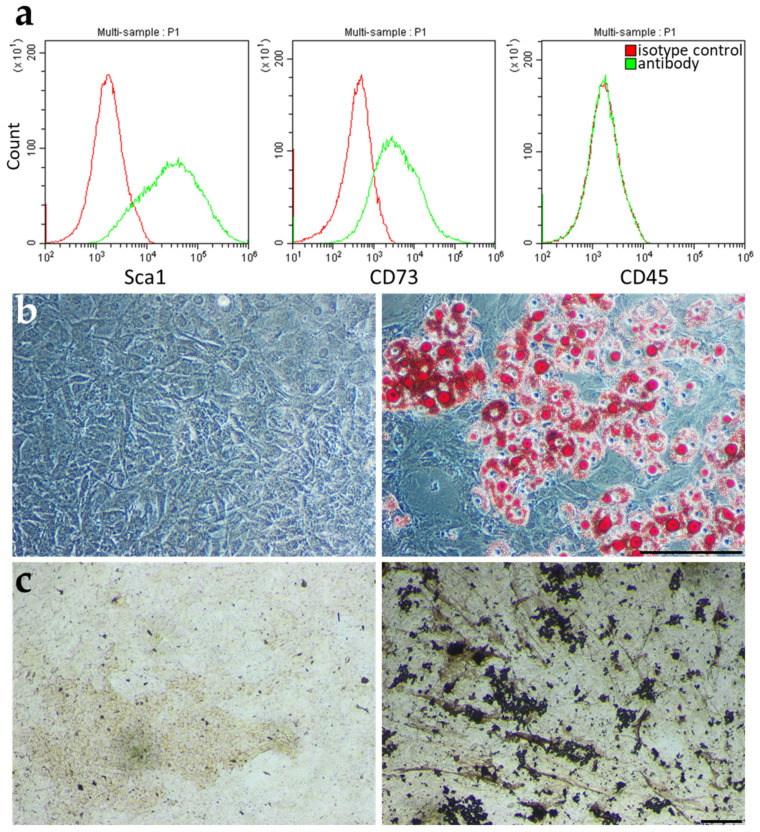
BALB/c MSC differentiate and express MSC specific markers. (**a**) Flow cytometric analysis of MSC cell surface markers (Sca1 and CD73) and of the pan-hematopoietic marker CD45. BALB/c MSC potential to differentiate into adipogenic (Oil Red O staining) (**b**) and osteogenic (Von Kossa staining) (**c**) cells. In (**b**,**c**) on the left are images of cells treated with control media while on the right are images of cells treated with differentiation media. Scale bar: 200 µm.

Treatment of experimental aGvHD used MSC derived from bone marrow (BM) of BALB/c mice, cultured for several passages, and characterized as previously detailed [20]. Briefly, MSC expression of specific markers was assayed for Sca1 and CD73 and absent expression of the pan-hematopoietic marker CD45 (Figure 1a).

The differentiation potential of the BM-derived MSC (BM-MSC) was evaluated for adipogenic (Figure 1b) and osteogenic (Figure 1c) lineages under corresponding culture conditions. Thus, the BM-derived cells from BALB/c mice present the characteristics of MSC.

**Figure 2:**
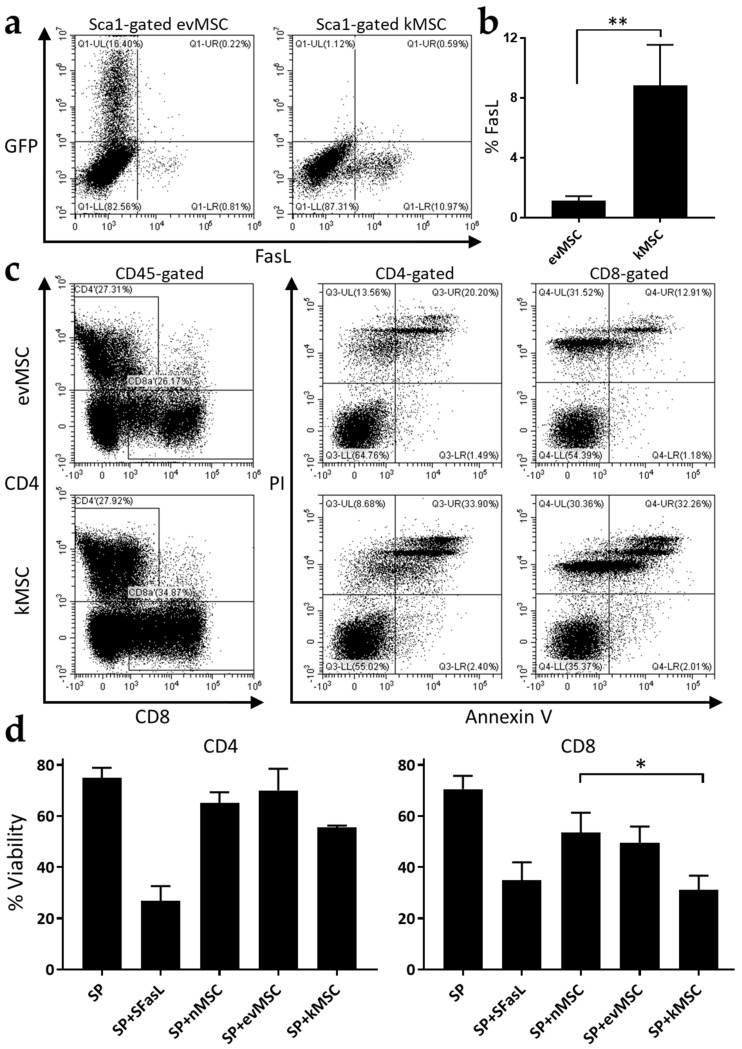
MSC overexpressing FasL induce the death of CD4^+^ and CD8^+^ T cells. (**a**) Representative flow cytometric dot-plots depicting GFP and FasL expression in MSC following transduction with Ad-GFP (**left**) and Ad-FasL-GFP (**right**). (**b**) Quantification of flow cytometric analysis of FasL expression on MSC transduced with the two adenoviruses. (**c**) Representative apoptosis analysis of activated splenocytes following co-culture with empty vector and FasL adenovirus-transduced MSC. In the dot-plots on the left are presented the gates for CD4^+^ and CD8^+^ cells (from the CD45^+^ population) while on the right are the dot-plots of PI- and Annexin-V-labelled CD4^+^ and CD8^+^ populations. (**d**) Graphical representation of the percentage of viable CD4^+^ and CD8^+^ cells after 72 h co-incubation with SuperFasL (SFasL), naïve MSC (nMSC), empty vector (evMSC), and FasL adenovirus-transduced MSC (kMSC). Data are means ± SD (*n* = 3), * *p* < 0.05, and ** *p* < 0.01.

The immunomodulatory properties of the MSC engineered to express FasL by adenoviral transduction were assessed using a moderate dose of 100 transduction units (TU)/cell established in preliminary studies. Adenoviral transduction caused an average eight-fold increase in numbers of MSC expressing FasL compared with the MSC transduced with empty vector (Figure 2a,b). Functional assays represented by a mixed inhibitory reaction in which CD3/CD28-stimulated splenocytes and MSC were co-cultured, showed that FasL-transduced MSC reduced the viability of both CD4^+^ (~10%, ~15%) and CD8^+^ T cells (~23%, ~19%) compared with naïve MSC and MSC transduced with empty adenoviral vector, respectively (Figure 2c,d). The levels of apoptosis for CD8^+^ T cells were in fact similar to splenocyte incubation with 100 ng/mL recombinant FasL protein (SuperFasL) (Figure 2d). These data demonstrate that overexpression of FasL in MSC, from here on referred to as killer MSC (kMSC), caused significant enhancement in T-lymphocyte inhibition and apoptosis.

### 2.2. Treatment with Killer MSC Reduces GvHD Severity and Extends Survival

**Figure 3:**
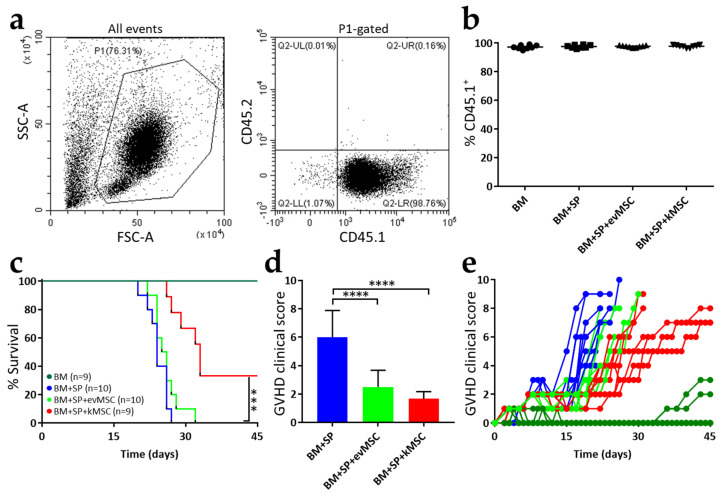
Treatment with kMSC improves GvHD clinical score and survival. Acute GvHD progression was studied in a model of lethal GvHD of haploidentical hematopoietic cell transplants induced by infusion of parental splenocytes into F1 chimeras. (**a**) Representative flow cytometric analysis of the engrafted cells (B6SJL, CD45.1^+^) in the recipient mice (F1, CD45.2^+^) at 3 weeks after transplant. (**b**) Quantification of engraftment in the transplanted mice. Each dot represents one mouse (*n* = 9 for BM, *n* = 10 for BM + SP, *n* = 10 for BM + SP + evMSC, and *n* = 9 for BM + SP + kMSC). (**c**) Survival curves for the experimental groups. (**d**) Graphical representation of the clinical score values 18 days after transplantation. (**e**) Evolution of the clinical score after transplantation. Each line represents one mouse. Error bars are means ± SD, *** *p* < 0.001, and **** *p* < 0.0001.

The impact of kMSC was assessed in a model of lethal aGvHD following haploidentical hematopoietic cell transplant induced by infusion of parental cells into F1 hybrids (H-2K^b^ → H-2K^b/d^). F1 recipients were conditioned with a lethal dose of X-rays and one day after irradiation they were grafted with a mixture containing 5 × 10^6^ bone marrow cells. In initial experiments, we tested incremental numbers of splenocyte infusions and chose a dose of 3 × 10^7^ cells/mouse that results in 100% GvHD-mediated death within 30 days post-transplantation (Figure S1). Three weeks after cell infusion, blood samples were harvested from the submandibular vein of the mice and the efficiency of the transplant was evaluated. Under our transplant conditions, all grafted F1 mice displayed >95% engraftment of parental CD45^+^ lineages (Figure 3a), irrespective of addition of MSC (Figure 3b).

All the mice that did not receive MSC died within 30 days and only one out of 10 mice infused with evMSC survived shortly over 30 days. A quite significant 33% improvement in survival was observed for mice infused with therapeutic kMSC (Figure 3c). Two of the three mice that survived for 45 days were still alive 100 days after transplant. GvHD progression was assessed using an established scoring system [21,22]. Mice were monitored daily, or every other day, and changes of weight, posture, activity, fur texture, and skin integrity were recorded and scored. All recipients of parental splenocytes displayed high GvHD scores, whereas MSC infusion delayed progression of GvHD symptoms and clinical scores (Figure 3d,e). However, after ~18 days there was an abrupt increase in clinical scores of mice infused with evMSC, which was slower in recipients of kMSC (Figure 3d,e).

**Figure 4:**
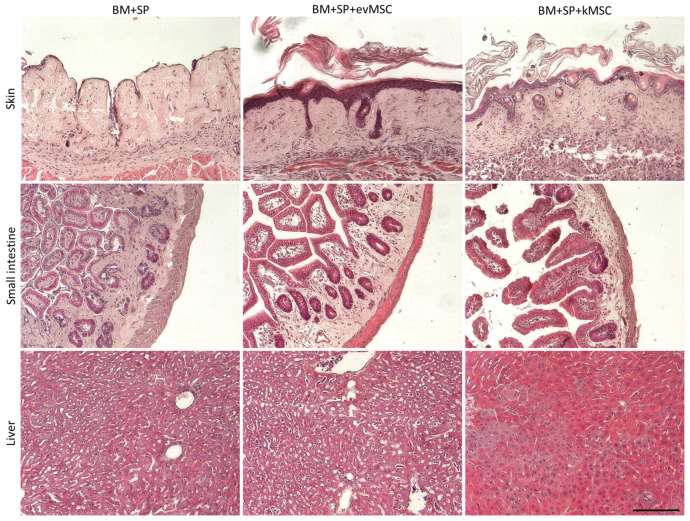
Mice treated with kMSC display reduced skin, small intestine, and liver histological damage. Hematoxylin and eosin staining of paraffin sections of skin (upper panels), small intestine (middle panels), and liver (lower panels) from the indicated experimental groups. Scale bar: 100 µm.

Tissue damage induced by GvHD and the influence of MSC treatment was assayed by histological analysis of the skin, small intestine, and liver (Figure 4). F1 mice from the different experimental groups were analyzed at 3 weeks post-transplant. The skin of untreated mice presented complete loss of follicles, thinned epidermis, and infiltrate surrounding follicular spaces as well as absence of adipose tissue. The skin from mice treated with evMSC had thick epidermis with a reduced number of follicles still present. Cellular infiltrates were observed at the basis of follicles and adipose layer was absent. Histological features of kMSC transplanted mice skin were the closest to normal, with increased number of follicles and presence of all dermal layers (Figure 4). Untreated mice displayed thickened small intestine wall with focal loss of mucosa and glandular destruction. No such defects were observed in the small intestine of kMSC-treated mice, while evMSC-treated mice showed intermediary glandular destruction and intestinal wall thickening (Figure 4). In the untreated mice, the small intestine was significantly shorter and the stomach was smaller. Treatment with kMSC seemed to partially rescue small intestine and stomach shrinking (Figure S2).

The liver sections of untreated and evMSC-treated mice looked similar presenting increased expansion of the sinusoids, perivascular mononuclear cells infiltration, and abnormal parenchyma with smaller hepatocyte nuclei, while no major abnormalities were observed in the livers of kMSC-treated mice (Figure 4). Histological analysis confirmed the improved status of the mice treated with kMSC.

Together, these results indicate a clear clinical advantage for the mice that received MSC treatment, with a more important improvement of the clinical status and survival for the mice treated with kMSC.

### 2.3. kMSC Treatment Induces a Decrease in CD25 Expression on Effector T Cells

**Figure 5:**
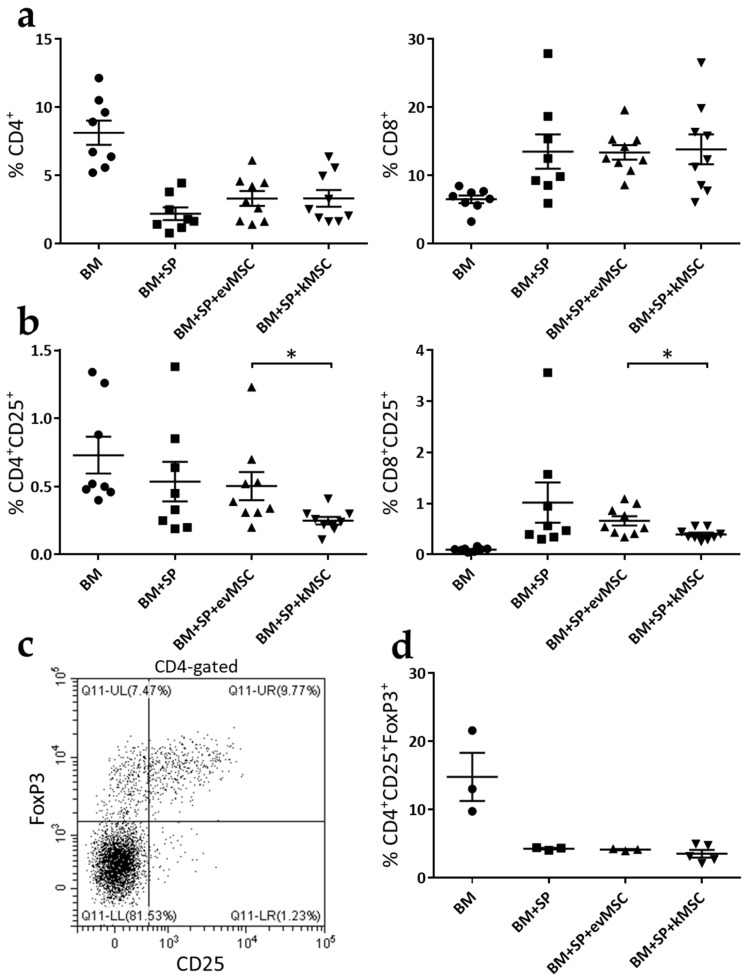
Treatment with kMSC results in lower CD25 expression on T cells, but not reduced numbers of T_reg_. Three weeks after transplant, mice were immunophenotyped. (**a**) Percentage of CD4^+^ and CD8^+^ cells in peripheral blood at 3 weeks after transplant. (**b**) Percentage of CD4^+^CD25^+^ and CD8^+^CD25^+^ cells in peripheral blood at 3 weeks after transplant. (**c**) Representative dot plot of labelled CD4^+^CD25^+^FoxP3^+^ cells from spleens of transplanted mice, 3 weeks after transplant. (**d**) Graphical representation of the percentage of CD4^+^CD25^+^FoxP3^+^ cells from spleens of transplanted mice. Each dot from the graphs represents data from one mouse. Error bars are means ± SEM, * *p* < 0.05.

To assess the in vivo effect of MSC treatment, mice were immunophenotyped 3 weeks after transplantation (Figure 5). First, we quantified the percentage of CD4^+^ and CD8^+^ cells from the peripheral blood. We did not find any significant decrease in the percentage of these cells that would explain the improvements of GvDH that we have observed in the mice treated with MSC (Figure 5a). We also evaluated the percentage of CD4^+^CD25^+^ and CD8^+^CD25^+^ in the peripheral blood and we observed an approximate two-fold decrease in the percentage of these cells for the mice treated with kMSC compared with those treated with evMSC (Figure 5b). It is known that CD25 is expressed on both activated effector T cells and Treg [23]. To distinguish if the decrease in CD25 expression reflects an effect of kMSC on the Treg compartment, we performed an additional transplantation experiment. Three weeks after transplant, we quantified the percentage of Treg (as CD4^+^CD25^+^FoxP3^+^ cells) from the spleens of the transplanted mice (Figure 5c) and we did not observe a significant decrease in the mice infused with kMSC compared with mice transplanted with evMSC or untreated mice (Figure 5d). These results suggest that the decrease in CD25^+^ T cells in the mice treated with kMSC represents a reduction in the number of activated T cells, which is further responsible for the improvements of GvHD recorded following kMSC treatment.

## 3. Discussion

We have previously shown that transduction of MSC to overexpress FasL improved their immunosuppressive activity in vitro, while maintaining the MSC patterns of marker expression and differentiation [20]. In this study, we assessed one of the potential applications of killer MSC in reduction of the severity of acute GvHD reactions.

Several studies have shown that naïve MSC hold the potential to suppress acute GvHD [24,25]. In our hands, naïve MSC only delayed the progression of the GvHD as seen in the clinical scores and histological analysis during the first 3 weeks post-transplant. Recent meta-analysis of the prophylactic and therapeutic efficiency of MSC in GvHD showed organ-specific responses, with superior activity in defense of the skin [7]. Our current data concur with these findings, with quite prominent difference in disease severity compared with the gastrointestinal tract and liver. For pragmatic purposes we refer to MSC transduced with empty vector as naïve MSC, as transduction itself did not affect the expression and differentiation patterns of these cells. The tissue-specific activity of MSC may originate from difficulties in navigation of these cells. However, MSC that reach circulation seem to be able to migrate within the tissues and reach the sites of active inflammation [26,27] where they perform their immunosuppressive activity through paracrine mechanisms.

Killer MSC further postponed the evolution of aggressive aGvHD, however, the impact of both subsets of MSC was rather transient and the immune disorder progressed to induce death of ~70% of the mice. It appears that the stringent haploidentical aGvHD model used here was too aggressive, and the therapeutic approach was insufficient to reduce disease severity to the extent of rescue of the recipients. The experimental model evidently affects the results, and might have been optimized to yield better results. Considering the wide range of GvHD severity in the clinical setting, we believe that current data provide a proof of concept to superior activity of MSC endowed with killing activity. In variance from functional suppression of GvHD effector cell activity by MSC, their elimination through Fas/FasL-induced apoptosis has indeed the capacity to suppress inflammation by reducing the inflammatory burden.

The model was determined according to several considerations. First, preliminary data showed reduced transduction efficacy of MSC from BALB/c mice, characterized by lower levels of coxsackievirus and adenovirus receptor (CAR) expression [22]. Second, the conditions of MSC transduction were experimentally tested, taking into account several considerations. MSC transduction with Ad-FasL-GFP at a ratio of 250 TU/cell was associated with significant cell death. At a transfection dose of 100 TU/cell, MSC viability was improved and cells displayed enhanced T-cell killing potential. These experimental conditions induced sufficient FasL expression to enforce T cell apoptosis, and was low enough to avoid suppression of MSC proliferation and differentiation [14]. Third, although mRNA expression of FasL was detected 8 h following adenoviral transduction [25], detection of the GFP reporter protein was highly variable. GFP expression in BALB/c-derived MSC was detected when the cells were transduced with more than 500 TU/cell [28]. Thus, FasL expression exceeded GFP almost eight times compared to cells transduced with the empty vector control adenovirus. It should be noted that FasL expression may be detected prior to protein export to the cell surface and therefore expression may be dissociated from the apoptosis-inducing function.

Immunophenotyping of the peripheral blood of treated mice revealed a two-fold decrease in CD25 expression in both CD4^+^ and CD8^+^ T cell populations in recipients of kMSC compared with evMSC. CD25 is a dual marker expressed on activated T cells as well as on Treg [23]. Analysis of the latter subset, according to joint expression of the FoxP3 transcription factor, showed stable Treg fractions in spleens of mice treated with kMSC, similar to those treated with control evMSC. Although MSC [5] have been associated with induction of Treg under selected experimental settings, it remains to be determined whether the particular combination of MSC and FasL abolishes the inductive activity. The evolving scenario indicates that the activity of FasL-expressing MSC in transient amelioration of GvHD severity occurred through suppression of effector cell activity.

This mode of action is consistent with previously reported depletion of putative effector cells using FasL protein ex vivo prior to transplantation, which indicates that a subset of sensitization-prone T cells is responsible for excessive morbidity caused by this immune reaction [29,30]. Modulation of GvHD has also been achieved by overexpression of FasL protein in T cells [31] and in vivo therapy with regulatory T cells endowed with killing activity by means of FasL overexpression [32]. In addition to the use of an extremely severe disease GvHD model, the modest results obtained here may be attributed to markedly superior navigation capacity of T cells compared with MSC to sites of inflammation [33]. Combination of pre-transplant exposure of the graft to FasL and administration of kMSC may provide a better therapeutic strategy for GvHD with cumulative effects and superior clinical outcomes.

## 4. Materials and Methods

### 4.1. Mice

The C57BL/6 (Stock No: 000664), BALB/c (Stock No: 000651), and B6-SJL (Stock No: 002014) mice were purchased from The Jackson Laboratory and grown in the institutional animal facility. All animals were maintained under specific pathogen-free conditions in a controlled environment with a 12 h shift of the light/dark cycle, 21 °C and 55–60% humidity, and had unrestricted access to food and water.

### 4.2. MSC Isolation and Culture

MSC were isolated from bone marrow extracted from femurs and tibias of 8-week-old BALB/c male mice. After being passed through a 40 µm strainer, the cell aspirate was pelleted and resuspended in MSC media containing: DMEM 1 g/L glucose (Thermo Fisher Scientific, Waltham, MA, USA), 10% fetal bovine serum (FBS) suitable for MSC culture (MSC-FBS, PAN-Biotech, Wimborne, UK) and 1% penicillin/streptomycin/amphotericin (PSA, Sigma, St. Louis, MO, USA). Cells were cultured starting with a 2 × 10^6^ cells/cm^2^ cell density and were left to adhere for 24 h; then, media were replaced every 2 to 3 days. Adherent cells were left to reach confluency and then subjected to several passages, each time at a 1:4 split. Cells were grown in a 37 °C, 5% CO_2_ humidified incubator. All the MSC in this study were used between passage 5 and 9.

### 4.3. Osteogenic and Adipogenic Differentiation of MSC

To determine the multipotency of BALB/c-derived MSC, cells were seeded at a 5000 cell/cm^2^ in 24-well plates and left to adhere overnight. Twenty-four hours after passage, media were changed with adipogenic and osteogenic induction media, respectively, as well as normal MSC media. Media were changed every 2 to 3 days. The adipogenic induction media contained DMEM 1 g/L glucose supplemented with 10% MSC-FBS, 1 µM dexamethasone, 100 mM indomethacin, and 1% insulin–transferrin–selenium (ITS-G, Thermo Fisher Scientific, Waltham, MA, USA), and the osteogenic induction media was based on DMEM 1 g/L glucose supplemented with 10% MSC-FBS, 0.1 µM dexamethasone, 10 mM β-glycerophosphate, and 0.3 mM ascorbic acid. After 2 weeks, cells were washed with phosphate buffer saline (PBS), fixed with 4% paraformaldehyde, and stained with Oil Red O to visualize the lipid droplets accumulated in the differentiated adipocytes or with von Kossa stain represented by incubation in 5% AgNO_3_, followed by a short 2 min rinse with 5% sodium thiosulphate to visualize the Ca^2+^ crystals. The images were taken using an Olympus CKX41 inverted microscope with an Olympus XC30 camera and minimally processed using Adobe Photoshop software, version CS3 (Adobe, San Jose, CA, USA).

### 4.4. Viral Transduction of MSC

MSC were detached by 0.125% trypsin-ethylenediaminetetraacetic acid (EDTA) (Sigma, St. Louis, MO, USA), washed and resuspended in DMEM 1 g/L glucose without sodium bicarbonate (Sigma, St. Louis, MO, USA) supplemented with 10% MSC-FBS at a density not larger than 2.5 × 10^6^ cells/mL. Maximum 200 µL cell suspension (5 × 10^5^ cells) were added in a 1.5 mL low-retention Eppendorf tube, followed by the addition of the corresponding amount of Ad-GFP or Ad-FasL-GFP virus [20]. The tubes were then stirred at 1000 rpm for 45 min at 37 °C on a thermomixer, without allowing the cells to sediment. Cells were seeded at corresponding densities, according to the following applications.

### 4.5. Flow Cytometry Analysis

All the reagents used in this study for flow cytometry were purchased from Biolegend (San Diego, CA, USA) unless otherwise specified. MSC were labeled with the following antibodies: anti-mouse Sca1-PE, anti-mouse CD73-PE/Cy7, and anti-mouse CD45-PE. Splenocytes were stained with anti-mouse CD45-PE, anti-mouse CD4-BV785, and anti-mouse CD8a-APC/Fire750 antibodies. Apoptosis of splenocytes was evaluated by flow cytometry after labeling the cells with PI and Annexin V-APC according to manufacturer’s protocol. Engraftment efficiency was tested with anti-mouse CD45.1-PE and anti-mouse CD45.2-APC. The antibodies used for peripheral blood immunophenotyping were: anti-mouse CD45.1-PE, anti-mouse CD3-PacificBlue, anti-mouse CD4-BV785, anti-mouse CD8a-APC/Fire750, and anti-mouse CD25-APC. Treg were identified following labeling of splenocytes with the same antibodies as for immunophenotyping and anti-FoxP3-AlexaFluor488. Prior to FoxP3 staining, the cells were fixed and permeabilized using True-Nuclear Transcription Factor Buffer Set according to manufacturer’s protocol. FasL was detected using anti-FasL-AF647 antibody (MLF4 clone, Bio-Rad, CA, USA). For all the antibodies, we used the corresponding isotypes from the same sources. Samples were analyzed using a CytoFLEX flow cytometer (Beckman Coulter, Indianapolis, IN, USA) and CytExpert software, version 2.1.

### 4.6. Immunosuppression Assay

Splenocytes used in these experiments were isolated from BALB/c female mice. Freshly harvested cells were resuspended in RPMI medium supplemented with 10% FBS, 1% PSA, and 50 µM β-mercaptoethanol (MLR medium) in the presence or absence of CD3/CD28 stimulation beads (in a ratio of 1:1 beads-to-splenocytes) (Dynabeads mouse T-Activator CD3/CD28, Thermo Fisher Scientific). Stimulated splenocytes were cultured alone, or in the presence of naïve, Ad-GFP- and Ad-FasL-GFP-transduced MSC (as above) in a ratio of 1:2 MSC-to-splenocytes for 72 h in the MLR medium. At the end of the co-incubation, splenocytes were carefully detached from the MSC by gentle rinsing using a 0.05 mM EDTA in PBS solution, stained with anti-CD45, -CD4, and -CD8 antibodies, PI and Annexin V, and analyzed by flow cytometry. SUPERFASLIGAND (SuperFasL) protein (Enzo Life Sciences, Farmingdale, NY, USA) at a concentration of 100 ng/mL was used as a control for FasL-induced apoptosis.

### 4.7. GvHD Mouse Model and Treatment

To determine the therapeutic effect of kMSC on aGvDH, we used a haploidentical parent to the F1 mouse (H-2K^b^ → H-2K^b/d^) model in which the bone marrow cells and aGvHD-inducing splenocytes from B6.SJL mice (congenic to C57BL/6) were transplanted into F1 mice obtained from breeding BALB/c females with C57BL/6 males. Thus, 9–11 weeks old F1 mice (males and females) were lethally irradiated with 8.5 Gy (Rad Source RS2000 Biological System) and 24 h later they were infused with 5 × 10^6^ whole bone marrow cells (control group—BM), 5 × 10^6^ bone marrow cells and 3 × 10^7^ splenocytes (aGvHD group—BM + SP) and bone marrow, and splenocytes and 1 × 10^6^ control evMSC or kMSC (MSC-treated groups—BM + SP + evMSC and BM + SP + kMSC). Bone marrow cells were sex matched for each transplanted mouse. We initially tested lethality progression with increasing doses of infused splenocytes (Figure S1). We chose to use for our model an infusion of 3 × 10^7^ splenocytes, a dose for which all untreated mice die within 30 days from transplantation. Monitoring of the mice was performed daily, or every other day, using an established aGvHD clinical grading system initially proposed by Cooke et al. [21] and modified by Schroeder et al. [22]. Weight loss, hunched posture, skin integrity, fur aspect, and activity were each assigned scores of 0 (absent), 1 (moderate), or 2 (severe). The sum of these scores represents the total GvHD clinical score with a maximum achievable value of 10.

### 4.8. Histology

Skin, small intestine, and liver samples were collected from mice belonging to each experimental group, 3-weeks after transplant. Mouse skin biopsies were collected from the interscapular region of the mice. The small intestine samples were collected from the 2 cm region above the cecum, while the liver tissue was harvested from the left lobes. The samples were fixed for at least 48 h in 4% paraformaldehyde and embedded in paraffin. The sections were stained with hematoxylin and eosin (H & E fast staining kit, Carl Roth, Karlsruhe, Germany) according to manufacturer’s recommendation. The images were captured using a Leica DMi8 inverted microscope with a Leica DFC9000 sCMOS camera and processed using Leica LAS X software, version 3.3 (Leica Microsystems, Wetzlar, Germany).

### 4.9. Statistical Analysis

Analysis of the data was performed using the GraphPad Prism program (GraphPad Software, San Diego, CA, USA). Data are presented as ± SEM or SD and were analyzed using the statistical test (Student’s *t* test or one/two-way ANOVA), suitable to each experiment. Log-rank test was used for Kaplan–Meier analysis of survival differences. Samples that had a *p*-value < 0.05 were considered statistically significant.

## Supplementary Materials

The following are available online at https://www.mdpi.com/article/10.3390/ijms23010534/s1.
