# Supplementary material for: Treatment with Mesenchymal Stromal Cells Overexpressing Fas-Ligand Ameliorates Acute Graft-versus-Host Disease in Mice
Source: Int J Mol Sci. 2022 Jan 4;23(1):534. doi: 10.3390/ijms23010534 (PMC8745472; doi:10.3390/ijms23010534)
Supplement: Supplementary file 1 [file ijms-23-00534-s001.zip › ijms-1534222-supplementary.pdf]

International Journal of *Molecular Sciences*

MDPI

Article

Treatment with Mesenchymal Stromal Cells Overexpressing Fas-Ligand Ameliorates Acute Graft-versus-Host Disease in Mice

Andrei Mircea Vacaru *, Ana-Maria Mazilu, Madalina Dumitrescu, Ioana Madalina Fenyo, Anca Violeta Gafencu and Ana-Maria Vacaru

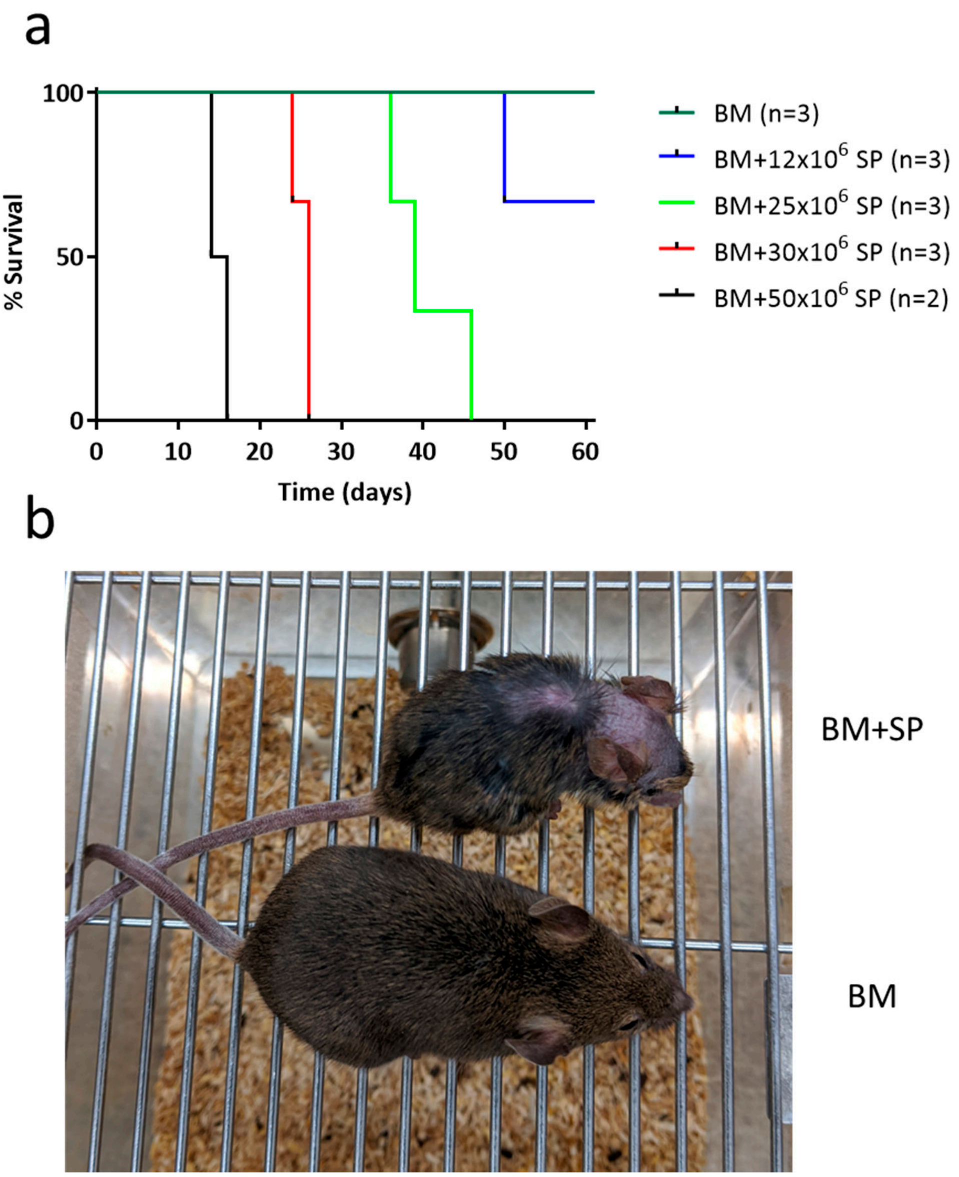

Figure S1. Higher doses of transplanted splenocytes accelerate aGvHD induced lethality. F1 mice were irradiated and cotransplanted with 5×10^6 haploidentical bone marrow (BM) cells and different doses of splenocytes (SP). The survival of mice transplanted with different numbers of splenocytes was monitored **(a)**. Mice transplanted with bone marrow and splenocytes presented clinical signs of aGvHD while those transplanted with bone marrow looked normal **(b)**.

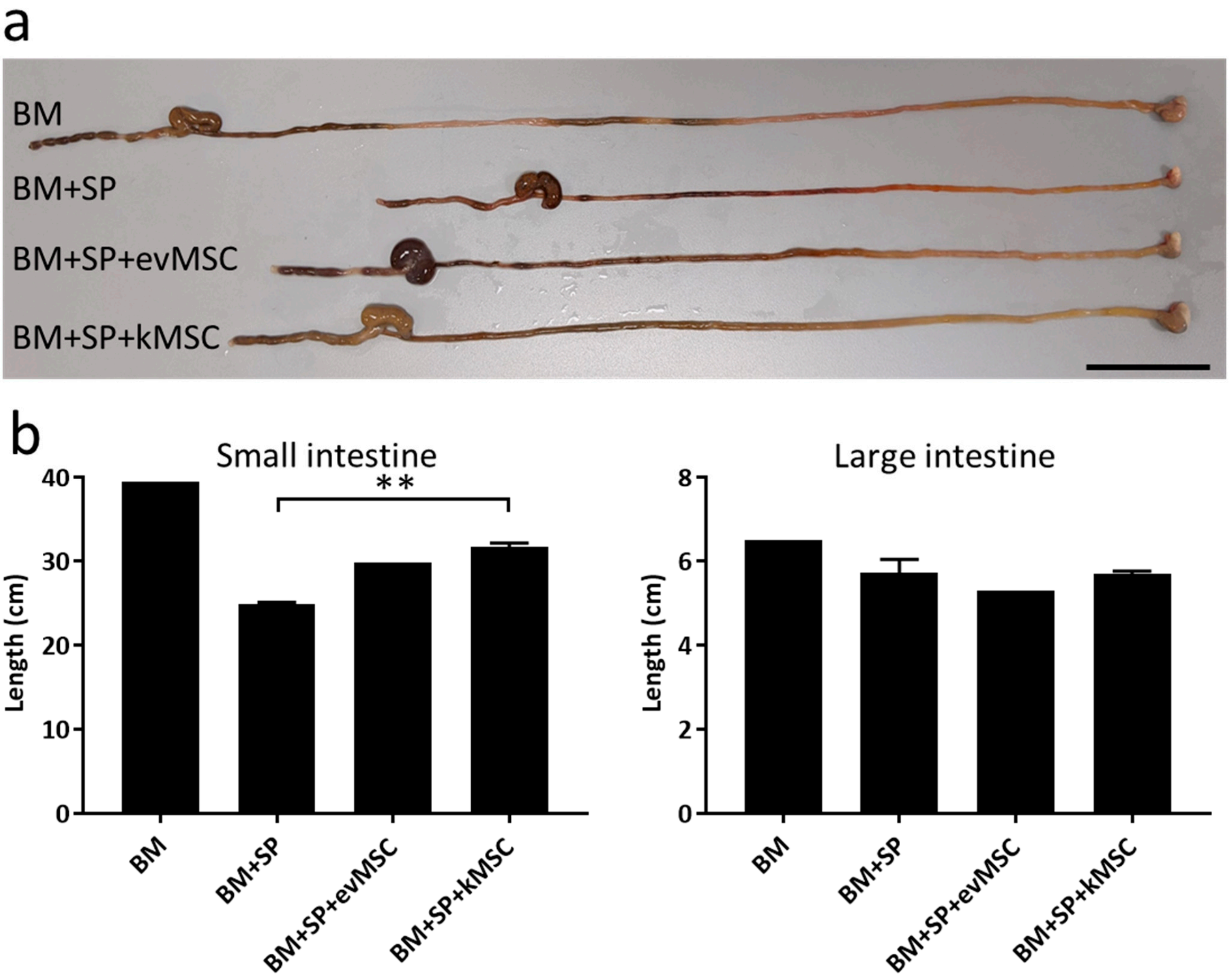

Figure S2. Mice treated with kMSC show reduced aGvHD-induced damage of the intestinal tract. Image of intestinal tract (from cardia to anus) of mice from each experimental group **(a)** and graphical representation of the measurements **(b)**. Data are means ± SD ($n = 2$), ** $p < 0.01$. Scale bar: 5cm.
